# Improving diabetic foot screening at a primary care clinic: A quality improvement project

**DOI:** 10.4102/phcfm.v8i1.955

**Published:** 2016-08-31

**Authors:** Michelle L. Allen, Albertine M.B. van der Does, Colette Gunst

**Affiliations:** 1 Division of Family Medicine and Primary Care, Stellenbosch University, South Africa; 2 Swartland subdistrict, Western Cape Government Health, South Africa; 3 Cape Winelands District, Western Cape Government Health, South Africa

## Abstract

**Background:**

Foot screening is an important part of diabetic care as it prevents significant morbidity, loss of function and mortality from diabetic foot complications. However, foot screening is often neglected.

**Aim:**

This project was aimed at educating health care workers (HCWs) in a primary health care clinic to increase diabetic foot screening practices.

**Setting:**

A primary health care clinic in the Western Cape province of South Africa

**Methods:**

A quality improvement project was conducted. HCWs’ needs were assessed using a questionnaire. This was followed by focus group discussions with the HCWs, which were recorded, transcribed and assessed using a general inductive approach. An intervention was designed based on common themes. Staff members were trained on foot screening and patient information pamphlets and screening tools were made available to all clinic staff. Thirty-two consecutive diabetic patient folders were audited to compare screening in 2013 with that in 2014 after initiation of the quality improvement cycle.

**Results:**

HCWs’ confidence in conducting foot screening using the diabetic foot assessment questionnaire improved markedly after training. Diabetic foot screening practices increased from 9% in 2013 to 69% in 2014 after the first quality improvement cycle. A strengths, opportunities, aspirations and results (SOAR) analysis showed promise for continuing quality improvement cycles.

**Conclusion:**

The findings showed a significant improvement in the number of diabetic patients screened. Using strategic planning with appreciative intent based on SOAR, proved to be motivational and can be used in the planning of the next cycle.

## Introduction

One in eleven people have diabetes, globally,^1^ and diabetes is a growing problem worldwide, with an estimated 642 million people suffering from the disease in 2040. In 2015, in South Africa more than 2.3 million people have been diagnosed with diabetes, and a further 1.3 million are undiagnosed. Prevalence in South Africa in 2015 was 7.6%.^1^ People with diabetes have a lifetime prevalence of developing a foot ulcer of about 15%.^2^ Marked morbidity, loss of function and mortality are associated with diabetic foot complications. More than half of lower extremity amputations in the United States are in diabetic patients,^3^ and amputations are more likely in poorer communities in the United States.^4^ This correlates with high rates of amputations in African countries.^5^ Diabetic foot ulcers result in 5-year mortality rates of 18–55% depending on the underlying cause of the ulcer.^6^ In 2009, it was estimated that diabetes in South Africa resulted in 78 900 years lost due to disability.^7^ Of these, 6% were due to amputations.^7^ The majority of foot complications can be prevented by appropriate diabetic management, foot care and footwear. Foot screening aims to detect, prevent and manage problems early in order to prevent many of the serious foot complications experienced by diabetic patients. International, national and local guidelines recommend foot screening to take place at least once a year for all diabetic patients.^8,9^

A study done in 2008–2009 in the Western Cape, South Africa, showed an increased prevalence of diabetes in the coloured community and a high prevalence of undiagnosed diabetes.^10^ The Society for Endocrinology, Metabolism and Diabetes of South Africa (SEMDSA)^9^ quotes a prevalence of type 2 diabetes in the coloured community of around 10.8%, which is supported by other studies conducted in similar communities.^11^

Klapmuts primary health care clinic, where the quality improvement cycle was done, serves a predominantly coloured community (more than 64%) within the Cape Winelands district (CWD) in the Western Cape, South Africa.^12^ As per international guidelines,^8,9^ the CWD includes annual comprehensive foot screening in its chronic disease management (CDM) plan and it is included in the CDM flowsheet developed in the CWD (see appendix 1).^13^ Implementation of guidelines in primary health care and subsequent behavioural change are influenced by many different factors. Passive dissemination has been found to be ineffective,^14^ and analysing the needs and obstacles within a target group has been shown to result in better adherence to guidelines.^15^

The annual integrated audit for chronic disease management^16^ done at Klapmuts clinic in 2013 showed that no diabetic patients had foot screening done according to current district and international guidelines. This finding generated the need for this quality improvement project. The aim was to improve diabetic foot care through increased diabetic foot screening practices by health care providers at the primary care clinic by conducting a quality improvement cycle. The foot screening tool that was used could be completed in 1–3 min by any healthcare providers at the clinic, including community workers, enrolled nursing assistants, staff nurses, professional nurses, doctors and the pharmacy assistant.

## Research methods and design

### Ethical considerations

Ethical approval was given by the health research ethics committee of Stellenbosch University (Reference No. S14/01/021). Permission to proceed with the study was obtained from the Western Cape Department of Health, the CWD Health Services and the operational manager of the facility. All of the staff who participated were fully informed and signed consent forms. The data obtained from patient folders were coded and no identifying data were used in the data collection process. The researcher was actively involved in the primary care clinic at the time, but no financial gain or conflict of interest was present.

### Study design

**FIGURE 1:**
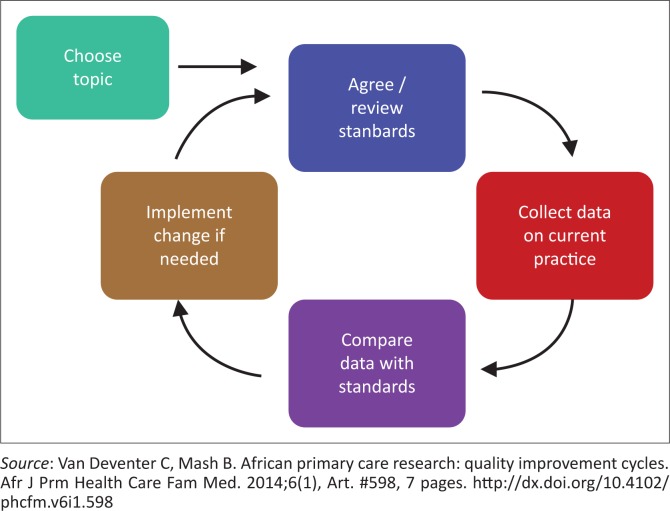
The quality improvement cycle.^18^

The HCWs at the clinic were involved in a quality improvement project using a quality improvement cycle.^17,18^ The steps followed in this quality improvement cycle are shown in Figure 1.

### Setting and population

The primary health care clinic is located in the Stellenbosch sub-district of the CWD. It serves a low- to middle-income population with a majority coloured population in a rapidly growing community of well over 12 000 people.^19^ The clinic is open every working day and manages approximately 2 400 patients per month. The clinic does not have a chronic disease register, but it is estimated that there are around 300–350 patients with diabetes at the clinic. Permanent clinic staff includes three clinical nurse practitioners, of whom one is the operational manager of the facility, one professional nurse, one staff nurse, one enrolled nursing assistant, one pharmacist assistant and one administration clerk. There are also two counsellors who work at the clinic, who are employed by a non-governmental organisation (NGO). There are several community care workers working in the community who are employed by the local hospice which is also an NGO.

### Selecting the team and designing the intervention

The clinic staff was a convenient sample to participate in the research, and all clinic staff members were invited to voluntarily take part in the quality improvement team. Team meetings or focus group discussions (FGDs) were facilitated by the researcher with a team of eleven participants including nursing staff, a community carer, the clerk, two counsellors and the pharmacist assistant. The qualitative data derived from the FGDs at team meetings were transcribed and analysed using a general inductive approach.^20^

**FIGURE 2:**
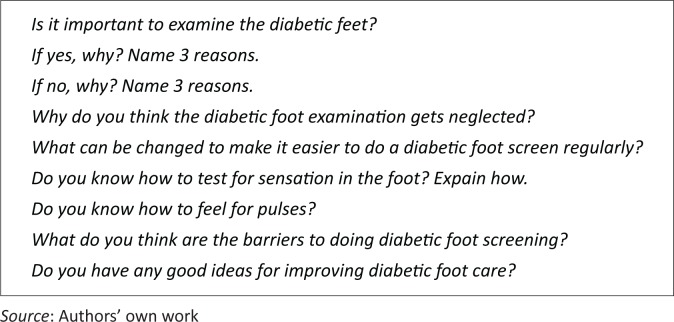
Klapmuts clinic staff questionnaire.

Prior to designing an appropriate intervention, the team completed a questionnaire (see Figure 2) to ascertain their knowledge and understanding of diabetic foot screening. The questionnaire was designed by the researcher and contained several open-ended questions, and these qualitative data were summarised and manually analysed by the researcher.

The Standards for QUality Improvement Reporting Excellence (SQUIRE) reporting guidelines for quality improvement were used as a guide.^21^

Prior to any intervention, the HCW questionnaire showed that the team had a good understanding of the importance of foot screening and the benefits thereof. For some of the clinical staff, there was uncertainty on exactly how to do the screening, for example how to test for sensation in the foot and where to feel for foot pulses. The team also considered various goals and barriers to foot screening as summarised in Table 1.

**TABLE 1:** Goals and barriers to foot screening summarised from health care workers questionnaire.

Goals	Barriers
Health promotion and patient education	Time constraints
Education and training of staff	Lack of importance attached to foot screen
Empowering patients	Regular staff shortages
Clear definition of staff roles	Patients’ reservation to have their feet examined
Diabetes Care Day	Foot hygiene
Opportunistic use of all visits to screen	Insufficient staff training and expertise

*Source*: Authors’ own work

During the FGD, the team discussed ideas for improving implementation of foot screening as well as education of staff and patients. They also anticipated potential difficulties in implementing certain changes regarding foot screening.

### Themes derived from the team meetings

The qualitative data from the FGDs were transcribed verbatim from the recording. Trustworthiness of the qualitative data was improved by triangulation with questionnaires, summary of findings presented to and verified by the team participants, and recordings of the FGD were kept for feedback from supervisor and an independent coder to compare themes using a general inductive approach.

The following five themes were identified from the team meetings/FGDs using a general inductive approach and two independent coders: patient education; health worker education; clinical care considerations; facility support and processes; equipment and stationery.

#### Patient education

Patient education needs identified by the team included: healthy lifestyle, effect of diabetes on feet, importance of good foot care, importance of foot screening, appropriate footwear and how to take care of their feet. The team suggested that the counsellors could be primarily responsible for group education to patients in the waiting room. Another place identified as a possible entry point for group education was the alternative distribution site where pre-packed medication is given to patients at the nearby community centre.

The team showed a lot of enthusiasm around the idea of organising a ‘Diabetic Day’ to be organised at the clinic with advertising and comprehensive diabetic education. This would be a long-term project. The available screen and video player are not used but were considered to be a possible route for education. The group also felt that every interaction with diabetic patients can be used for patient education and potentially for foot screening, that is, not just chronic visits.

#### Health care worker education

A need for expanding the number of people trained to do foot screening was discussed by the group. All health care staff, including counsellors in the clinic and carers in the community, should be trained to provide patients with information on diabetic foot care. All clinical nursing staff should be able to do a comprehensive clinical examination of a diabetic patient’s feet.

#### Clinical care considerations

The potential for patient embarrassment regarding foot odour, foot care or problems was considered. Potential solutions discussed included having foot cleaning facilities available; providing patients with disinfectant spray and paper towels to clean their feet; gloves for staff protection; adequate patient preparation, for example, patients coming to the clinic prepared because they know it is time for a foot screening; and supportive staff attitudes towards patients.

Triage was considered a good entry point for addressing foot screening needs and screening for undiagnosed diabetes. Although a busy triage area was not considered the place for conducting the foot screening, the group felt that it could be a place to identify patients requiring a foot screen by attaching the screening questionnaire to the front of the patient’s folder.

#### Facility support and processes

Improved triage functioning with a junior and senior staff member at all times was considered. The clinic’s high workload and disruption due to building at the time of the project, with upheaval of the filing area, staffing challenges and long hours, were discussed. Recruiting community health workers was identified as an avenue that needs further exploration. Staff training was to be done by the family medicine registrar at the clinic.

#### Equipment and stationery (structure)

A list of stationery required for foot screening can be seen in Table 2. Latex gloves and monofilaments are readily available at the clinic. The use of stickers on folders to more easily identify patients needing foot screening was considered in the group but will need discussion with affected patients.

**TABLE 2:** List of stationery requirements as discussed during focus group discussions comparing availability and use before and after intervention.

Stationery	Available and used prior to intervention	Available and used after intervention
Patient education leaflets	No	Yes
Foot screening questionnaires	Yes (not used)	Yes
Chronic disease flow sheets	Yes	Yes
Chronic disease register	Yes (not used)	Yes (not used)
Educational posters	No	No

*Source*: Authors’ own work

#### Setting standards and targets

During the team meetings the following standards were set:

- Foot screen done, proven by having a diabetic foot assessment questionnaire (DFAQ) completed in the patient folder
- CDM flow sheet included in folder
- Targets were set by the clinic team
- A 30% improvement of foot screens done before and after the intervention
- All files to include a CDM flow sheet

### Intervention

**FIGURE 3:**
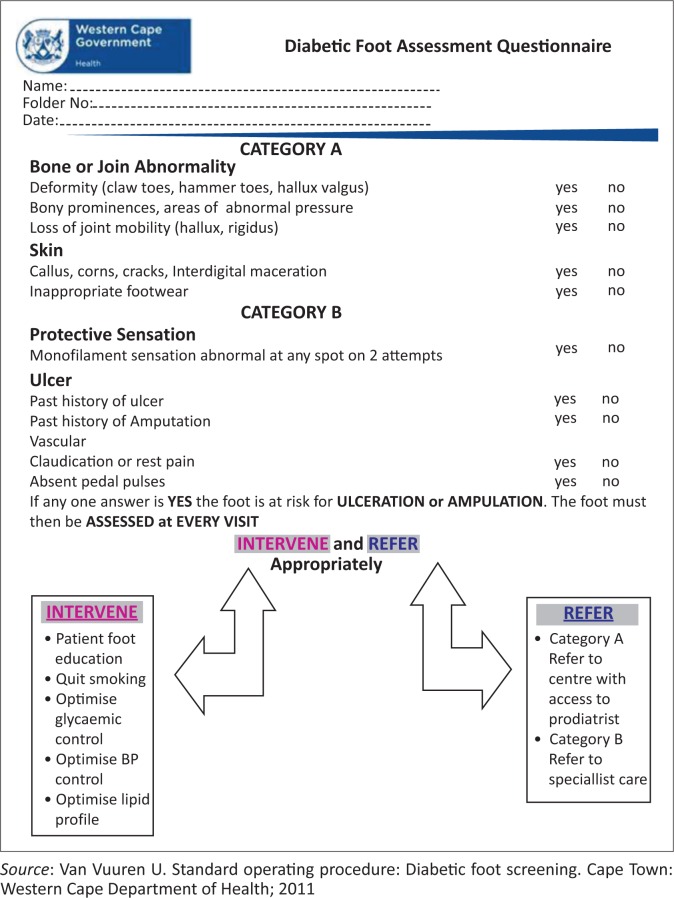
Diabetic foot assessment questionnaire developed by the Western Cape Department of Health.

The intervention was planned based on information gathered from the HCW questionnaires and the FGDs. The initial intervention involved staff training in foot screening technique and using the DFAQ developed by the provincial office of the Western Cape^22^ (see Figure 3).

This was done in small groups with each person being given an opportunity to show the others the full foot screening. Trouble-shooting was done during training. Education on the management of common diabetic foot ailments, foot care tips and footwear requirements were also given to the staff. The clinical staff was provided with a flip-file containing patient information leaflets in different languages. These leaflets were designed by the researcher combining educational resources from different tools.^22,23,24^ Monofilaments were available in all consulting rooms as well as the triage area. One of the counsellors, who volunteered to address groups of patients on the importance of foot screening, was given additional training in this regard by the researcher.

### Data collection

Three months after the intervention, 32 consecutive folders of diabetic patients were collected at the pharmacy over a period of 1 week. They were audited using the standards set by the team. All diabetic files were consecutively sampled, regardless of foot complications. In order to show a 30% improvement, it was estimated that 32 files were needed to be audited out of a total estimated 300 diabetic patients at the clinic.

The binomial sign (‘exact’) test was used and a *p*-value of < 0.01 would be considered significant.

### Data analysis

The collected data were used to assess change in the practice of foot screening by comparing results for 2013 to those for 2014 in the same folder. The researcher and an assistant trained in data collection captured the data in a spreadsheet, for analysis by the researcher and a statistician.

## Results

**FIGURE 4:**
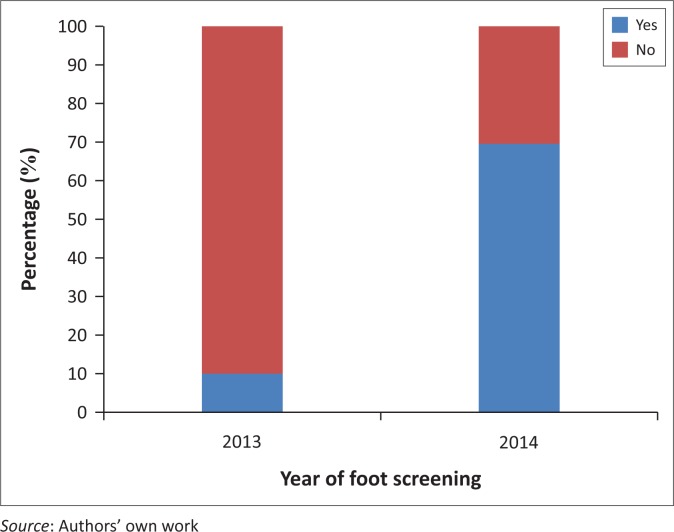
Foot screening completed in 2013 and 2014 (total of 32).

Thirty-two consecutive folders were audited, and all contained the CDM flow sheet. There was a marked increase in the number of patients who had undergone foot screening from 2013 to 2014 (see Figure 4). Apart from looking at the CDM flow sheet, clinical notes were also evaluated to look for evidence that a foot screening had been done. Any documentation on foot screen was counted as ‘foot screen done’. No DFAQs were found in the files for 2013.

### Evaluation

A feedback session was held with the team, where the results of the audit were given, to gain insight into their experiences as well as possibilities for future improvement planning. A synthesis of this feedback session was made focussing on SOAR analysis.^25^ The results are summarised in Table 3.

**TABLE 3:** SOAR analysis of feedback given by health care workers (HCWs) after the intervention.

Variable	Statements
Strengths	The project motivated the team
	Training of staff helped to build confidence
	Patient information pamphlets empowered staff to improve patient education as well as the patients to understand their illness and take responsibility.
	Revised foot screening questionnaire with pictures was found very helpful
	Folder in each consulting area with above resources available
	Necessary equipment readily available (monofilament, latex gloves, alcohol swabs)
	Clinic has a social media group for staff, improving communication and support
Opportunities	World Diabetes Day Event 14 November
	Educating HCWs and patients at alternative distribution site and school
	Training community health workers who are involved in home delivery of medication to patients who are less mobile.
	Systems improvement:
	Triage system to include more experienced clinical staff
	Appointments to be made in the afternoons to improve management of acute cases in morning
	Re-initiation of club days, e.g., booked diabetic patients to come on Tuesdays
	Improving efficiency and morale at the clinic
	Foot screening questionnaire to be in all diabetic patient folders
	Expanding social media group to include patients
	Getting appropriate educational material and fix video player
Aspirations	Further educating and empowering patients
	Continuous mindfulness of foot care and the on-going shared health improvements with patients’ involvement
	Yearly screening of hypertensive and patients at risk for diabetes
	All diabetic patients to have a minimum of an annual foot examination
	To run an annual World Diabetes Day programme in the community on 14 November
Results
	Provisional improvement of foot screening from less than 10% in 2013 to nearly 70% in the first half of 2014.
	Feedback from staff showed that their confidence in conducting foot screening as well as their enthusiasm for foot screening and patient education were much improved.
	Barriers were reduced by staff and patient education. All the clinical staff were trained and competent in foot screening as per the DFAQ. Patients were educated in the importance of foot care; few were unwilling for foot screening to be done.

*SOAR*, strengths, opportunities, aspirations and results; *DFAQ*, diabetic foot assessment questionnaire.

*Source*: Authors’ own work

## Discussion

A dedicated group of health care providers can be empowered to embark on quality improvement journeys. Despite challenges, this quality improvement project showed marked improvement in foot screening practices by HCWs at a primary health care clinic. Combined with strategic inquiry and appreciative intent, with a focus on the positive aspects of the process and future potential, the team can build on positive experiences, even during challenging times.

There was an improvement of foot screening from less than 10% in 2013 to nearly 70% in the first half of 2014. Feedback from staff showed that their confidence in conducting foot screening as well as their enthusiasm for foot screening and patient education were much improved.

A study in Uganda on patients with diabetic foot complications found that in terms of their beliefs about health and knowledge on foot care and self-care, education was urgently needed.^26^ In Klapmuts, the FGD with clinic staff raised the important theme of patient education, with several suggestions to enable better education and foot care. Foot self-care education has been shown to improve foot care and reduce diabetic foot complications.^27^ Similar studies using quality improvement cycles have shown similar results.^28,29^ One of the many strengths of this approach is the ability for rapid implementation of consecutive or concurrent cycles to keep quality improvement an ongoing process. Some of the studies had slightly more conservative results, for example, improving foot examination from 40% to 64%.^30^ This may be due to the larger group involved and individual team variables.

A consecutive sample of patient folders was used due to time constraints. Despite the possibility of selection bias, the results showed overwhelming improvement, and future randomised sampling should verify this success and improve internal validity.

A target of improving foot screening practices by 30% was set by the team. The need for a chronic disease register and a more accurate estimate of the number of diabetic patients cared for by the clinic will help with more precise power calculations for future studies.

The staff may have been more vigilant regarding foot screening during the time of data collection. A retrospective consecutive sample to be collected without the knowledge of the clinical staff was deemed impossible.

This relatively small quality improvement project showed a significant improvement in foot screening practices with simple and time-efficient interventions. The long-term benefit of improved diabetic foot care and reduced morbidity and mortality are beyond the scope of this study. Thus far, the staff’s enthusiasm towards foot screening and practice thereof has increased. This resulted in the increased number of patients having had foot screening. Due to the dichotomous nature of the data collection (either done or not done), the quality and completeness of the foot screening were not assessed.

Although each primary health care clinic has a unique team of HCWs and working circumstances, there are marked commonalities which should mean that generalisability is possible. Many of the goals and barriers will be similar to those at other clinics. Useful equipment for foot screening will be the same, and the need for education of patients and HCWs would, to varying degrees, be required.

Current evidence of a reduction in diabetic foot complications (ulcerations, infections and amputations) from using the diabetic foot screening questionnaire is limited.^30^

Qualitative research techniques to better understand the diabetic patients understanding and perceptions relating to foot screening and foot care could add valuable insight into future quality improvement planning.

Staff limitations and barriers identified were reduced through empowering staff with education, upskilling and understanding of the importance of foot screening.

### Recommendations

The recommendations are briefly listed as ‘Opportunities’ and ‘Aspirations’ in Table 4. Organising an event on World Diabetes Day on 14 November 2014 was enjoyed by staff and patients alike, providing a positive atmosphere and community engagement. The staff, especially the counsellors, should continue to provide group as well as individual counselling to the diabetic patients.

**TABLE 4:** Standards before and after intervention (*n* = 32).

Standards	2013	2014
Number of folders audited	32	32
Foot screen done	3	22
Foot screens with DFAQs in folder	0	17
CDM flow sheets in folder	32	32

*DFAQ*, diabetic foot assessment questionnaire; *CDM*, chronic disease management.

*Source*: Authors’ own work

A target of 100% of diabetic patients to have foot screens by 2014’s CDM audit is expected to be reached. Auditing of diabetic patient folders should be done quarterly, unexpectedly and randomly to improve internal validity and encourage sustainability, ongoing feedback and strengthening of the effort of the clinic team. The integrated CDM audit takes place annually and will be a way of tracking whether the improvements seen in this study are sustainable, annual foot examinations for diabetics being one of the indicators in the audit. It will also direct future quality improvement projects.

Ongoing quality improvement efforts are essential to sustain positive changes and staff motivation, and are in line with the Western Cape’s provincial focus on clinical governance.^31^ Shifting the focus from a traditional problem-based, deficit-based approach to a strength-based approach requires ongoing training and practical experience. Sustainable implementation requires support from health managers.

## Conclusion

This quality improvement project aimed at HCWs has dramatically improved diabetic foot screening at this clinic. The results showed significant improvement in foot screening practices by the HCWs. This study sets the benchmark of what may be possible and the lessons learnt will be very important for future evaluations.
